# Structure of Crenezumab Complex with Aβ Shows Loss of β-Hairpin

**DOI:** 10.1038/srep39374

**Published:** 2016-12-20

**Authors:** Mark Ultsch, Bing Li, Till Maurer, Mary Mathieu, Oskar Adolfsson, Andreas Muhs, Andrea Pfeifer, Maria Pihlgren, Travis W. Bainbridge, Mike Reichelt, James A. Ernst, Charles Eigenbrot, Germaine Fuh, Jasvinder K. Atwal, Ryan J. Watts, Weiru Wang

**Affiliations:** 1 Genentech, Inc., 1 DNA Way, South San Francisco, California 94080, USA; 2 AC Immune SA, EPFL Innovation Park, Building B, 1015 Lausanne, Switzerland

## Abstract

Accumulation of amyloid-β (Aβ) peptides and amyloid plaque deposition in brain is postulated as a cause of Alzheimer’s disease (AD). The precise pathological species of Aβ remains elusive although evidence suggests soluble oligomers may be primarily responsible for neurotoxicity. Crenezumab is a humanized anti-Aβ monoclonal IgG4 that binds multiple forms of Aβ, with higher affinity for aggregated forms, and that blocks Aβ aggregation, and promotes disaggregation. To understand the structural basis for this binding profile and activity, we determined the crystal structure of crenezumab in complex with Aβ. The structure reveals a sequential epitope and conformational requirements for epitope recognition, which include a subtle but critical element that is likely the basis for crenezumab’s versatile binding profile. We find interactions consistent with high affinity for multiple forms of Aβ, particularly oligomers. Of note, crenezumab also sequesters the hydrophobic core of Aβ and breaks an essential salt-bridge characteristic of the β-hairpin conformation, eliminating features characteristic of the basic organization in Aβ oligomers and fibrils, and explains crenezumab’s inhibition of aggregation and promotion of disaggregation. These insights highlight crenezumab’s unique mechanism of action, particularly regarding Aβ oligomers, and provide a strong rationale for the evaluation of crenezumab as a potential AD therapy.

Alzheimer’s disease (AD) is the most common form of dementia, affecting an estimated 5.3 million individuals in the United States and 46.8 million people worldwide^1^,^2^. The deposition of extracellular insoluble amyloid plaques composed primarily of amyloid-β (Aβ) peptides in the brain is a hallmark pathologic finding in AD^3^. An imbalance in the production and/or clearance of Aβ in brain leads to amyloid accumulation and is causally associated with AD pathogenesis^4^. The accumulation and aggregation of Aβ peptides in brain takes many forms in addition to plaques, including soluble monomers and oligomers, and insoluble fibrils, with a large range of molecular weights from 10 to 1,000 KDa (see review ref. 5). *In vitro* and *ex vivo* evidence suggests that soluble oligomers, i.e. “toxic Aβ oligomers,” may be primarily responsible for neurotoxicity^6^,^7^,^8^. Neutralization of toxic Aβ peptides (in its multiple forms) by anti-Aβ monoclonal antibodies is being pursued as therapies for AD, as growing evidence suggests passive immunization against Aβ can provide clinical benefit and perhaps AD prevention^9^.

Crenezumab is a fully humanized immunoglobulin isotype G4 (IgG4) anti-Aβ monoclonal antibody designed to bind multiple forms of Aβ (monomers, oligomers, fibrils and plaques). *In vitro* studies demonstrated crenezumab’s ability to block Aβ aggregation, promote Aβ disaggregation of oligomers, and protect neurons from oligomer-induced cytotoxicity^10^. This broad binding profile and novel mechanism of action, particularly regarding Aβ oligomers, suggest a potential for therapeutic efficacy. Crenezumab’s IgG4 backbone confers reduced activation of FcγRs in comparison to IgG1 and was shown to minimize FcγR-mediated inflammatory activation of microglia – which has also been proposed to contribute to neurotoxicity^11^,^12^ – while preserving FcγR-mediated microglial phagocytosis of oligomers^10^. In recent AD clinical trials involving monoclonal antibodies that bind aggregated forms of Aβ with IgG1 backbones that have fully preserved FcγR-mediated effector function, amyloid-related imaging abnormalities (ARIA) suggestive of vasogenic edema or effusions (ARIA-E) and microhemorrhage (ARIA-H) have been reported^13^. Crenezumab was designed as an IgG4 based on the hypothesis that an antibody with reduced effector function would have a lower risk of inducing ARIA-E/H and potentially provide a safety advantage over monoclonal antibodies that bind aggregated forms of Aβ with IgG1 backbones^14^. No increase in ARIA-E was observed with crenezumab in a Phase I study following either a single dose (0.3–10 mg/kg IV) or multiple (four) ascending weekly doses (0.5–5 mg/kg IV)^10^,^15^.

A number of monoclonal anti-Aβ antibodies have been tested in clinical studies (see review ref. 16). These antibodies target one or more of three classes of epitope; (1) aducanumab^17^, bapineuzumab^18^ and GSK933776^19^ recognize the N-terminus of Aβ; (2) solanezumab^20^ and crenezumab^10^ recognize the mid-region of Aβ; (3) ponezumab^21^ recognizes the C-terminus of Aβ. Gantenerumab^22^ recognizes an epitope that includes both amino acids from the N-terminus and mid-region. These antibodies display diverse preferences in engaging Aβ aggregates. Aducanumab and gantenerumab bind primarily to aggregated Aβ, whereas solanezumab is selective for soluble monomers. In contrast, bapineuzumab and crenezumab bind with high affinity to oligomeric forms. Coarse epitope classification of the antibodies does not correlate with their binding profiles. Not surprisingly, high-resolution X-ray crystal structures of antibody/Aβ complexes reported in recent years have revealed diverse epitopes^21^,^22^,^23^,^24^,^25^. It is also important to understand the Aβ peptide structure, especially in aggregates, where Aβ oligomerization can produce various products^26^,^27^,^28^,^29^,^30^. The basic building block appears to be a single Aβ peptide folded into a U-shaped hairpin-like structure. Hydrophobic residues, including Phe19 and Phe20 and Ile34, bring two strands together and a salt-bridge between residues Asp23 and Lys28 stabilizes the hairpin bend^28^,^31^,^32^,^33^,^34^,^35^ and consequently promotes nucleation of oligomers^36^. Murakami *et al*. reported a conformation-specific antibody (11A1) recognizing the hairpin bend structure around Glu22/Asp23 and binding to low-molecular weight oligomers^37^.

To our knowledge, crenezumab remains the only antibody that targets the mid-region of Aβ peptide and binds to multiple aggregated forms with dissociating effects. To gain insight into this ability, we performed an X-ray crystallographic study on the antibody/Aβ complex. In this report, we describe the structural result and follow up with nuclear magnetic resonance (NMR), mutagenesis and electron microscopy (EM) studies it inspired. Our results provide a precise molecular portrait and uncover critical elements giving rise to the unique functional modality.

## Results

### Crenezumab captures Aβ peptide in an extended conformation

The antigen-binding fragment (Fab) of crenezumab (CreneFab) was over expressed in *E. coli*. Co-crystallization of CreneFab with full length Aβ peptide was attempted but hindered due to the low solubility of Aβ peptides. Both Aβ_1–42_ and Aβ_1–40_ heavily precipitated in the aqueous environment conducive for Fab crystallization. We then examined a variety of epitope-containing^10^ fragments of Aβ peptide in co-crystallization trials. The truncated peptides generally showed improved solubility. The fragment with residues 11–25 yielded crystals diffracting to 2.3 Å resolution. For comparison, we also determined the crystal structure of CreneFab alone at 2.5 Å resolution.

**Figure 1:**
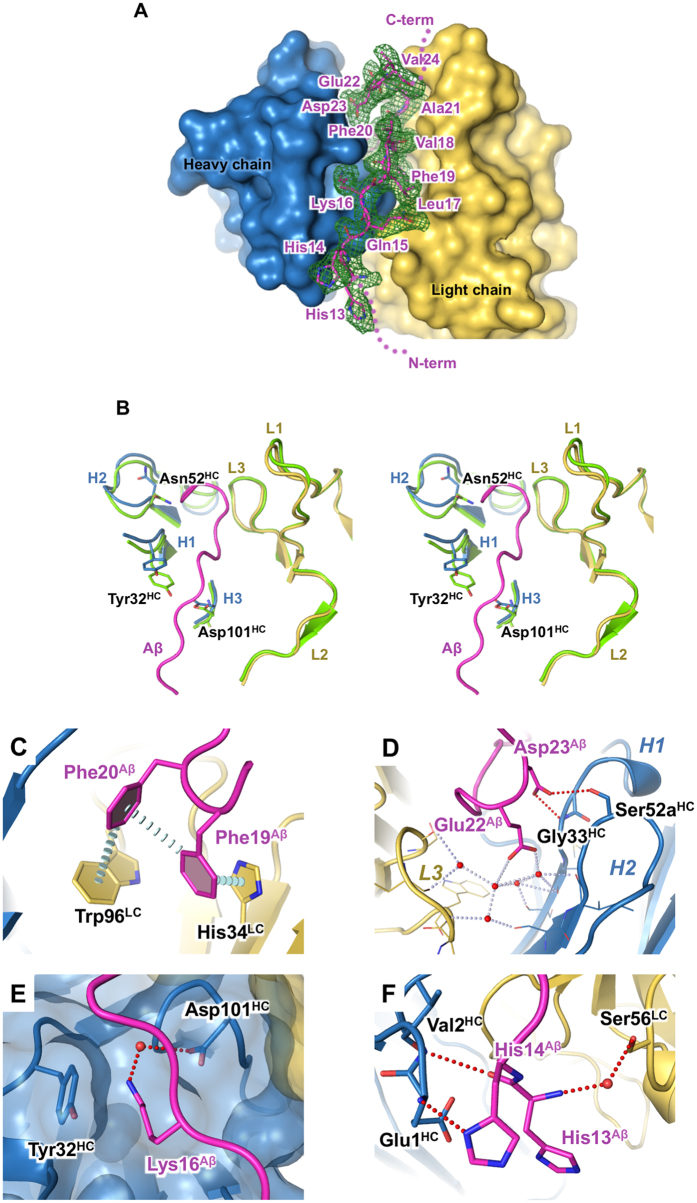
Crystal structure of CreneFab/Aβ. (**A**) The overall view of Aβ_11–25_ binding to the antibody. CreneFab is shown in surface rendering. Blue, heavy chain; Yellow: light chain. Aβ peptide is shown in ribbon and sticks. Carbon atoms of Aβ peptide are in magenta; other atoms are colored by atom-type. The N- and C-termini of Aβ peptide are disordered in structures as indicated by dots. The Aβ residues are labeled. Green mesh shows the 2Fo-Fc electron density map (contoured at 1xRMSD) corresponding to the Aβ peptide. (**B**) In side-by-side stereo view, shows an overlap of the CDR region from the Fab alone structure (green) with the Fab/Aβ complex (blue: heavy chain, Yellow: light chain), denoted H1, H2, H3, L1, L2, L3. (**C**–**F**) Close-up views of the binding site. Details are described in the results. Color scheme is same as in (**A**).

Crenezumab binds to a consecutive stretch of twelve Aβ_11–25_ peptide residues in an extended conformation (Fig. 1A). Aβ residues His13^Aβ^–Val24^Aβ^ adopt a well-defined structure, while the flanking residues on the N- and C-termini of the peptide are disordered as indicated by poor electron density. The crystallography epitope, therefore, comprises residues 13 through 24, which is consistent with previous epitope mapping results using enzyme-linked immunosorbent assay (ELISA) by Adolfsson *et al*.^10^. We further confirmed the ELISA epitope in this work using Ala or Gly substitutions shown in Figure S1.

The complementarity determining regions (CDRs) of crenezumab feature a very short H3 loop of 3 residues and a long L1 loop of 16 residues, thereby creating a deep paratope groove at the interface between V_H_ and Vκ. The Aβ peptide was engulfed in the groove in the complex structure. Interestingly, superposition of the CreneFab-alone and Aβ complex structures showed no major changes in the CDR structure. The root mean square deviation (RMSD) of Cα atoms for the variable domains and CDRs-only was 0.73 Å and 0.71 Å, respectively (Fig. 1B). More pronounced changes were observed in CDR residues Tyr23^HC^, Asn52^HC^ and Asp101^HC^, which adopted alternative side chain rotamers upon Aβ binding.

The Aβ peptide made extensive interactions with CreneFab, burying 773 Å^2^ of surface area on the antibody, most of which involved CDR residues. The shape complementarity score, S_c_^38^, of 0.74 is consistent with tight binding. We tested this empirically by measuring the crenezumab binding affinity to Aβ using surface plasmon resonance (SPR). As described in the methods section, it was technically challenging to control and maintain the density of immobilized Aβ on SPR chips. To generate monomer Aβ chips, we used fragments of Aβ peptide that encompass the epitope and are more soluble. We tested a variety of Aβ fragments with different N- and C-termini and observed similar affinity to crenezumab. The experimental variation resulted in a range of affinity values, which are considered to be equal within a reasonable margin. Figure S2 shows representative sensorgrams. The full-length IgG4 exhibited a K_D_ in range of 3.0–5.0 nM to Aβ monomers and 0.4–0.6 nM to Aβ oligomers, consistent with the structural prediction for high affinity interactions. The aromatic side chains of Phe19^Aβ^ and Phe20^Aβ^ formed a π-π stacking network comprised of one “face-to-face” and two “face-to-edge” interactions with light chain residues Trp96^LC^ and His34^LC^ (Fig. 1C). This π-π stacking network anchored the Aβ peptide to the bottom of the paratope groove. Toward the C-terminal end of the Aβ epitope, two negatively charged residues, Glu22^Aβ^ and Asp23^Aβ^, engaged in hydrogen bonds with Ser52a^HC^ and Gly33^HC^, while Glu22^Aβ^ was also linked to CDRs H1, H2, and L3 through a cluster of five structural waters (Fig. 1D). Another major site of interaction was around Lys16^Aβ^ and involved CDR H1 and H3 loops (Fig. 1E). This region of the antibody underwent relatively large structural changes upon Aβ binding, where the Tyr32^HC^ hydroxyl group shifted by ~3 Å, and Asp101^HC^ adopted an alternative rotamer conformation. The positive charge associated with Lys16^Aβ^ was neutralized by Asp101^HC^ as both side chains were sequestered at the binding interface and shielded away from bulk solvent. In addition, Lys16^Aβ^ formed a cation-π interaction with Tyr32^HC^.

We observed a non-canonical antibody-antigen interaction between Aβ and the N-terminus of the heavy chain (Fig. 1F). The His14^Aβ^ side chain formed a hydrogen bond with the terminal amino group of Glu1^HC^. An unprotonated state for His14^Aβ^ was implicit in this interaction and was consistent with the basic pH of crystallization (pH 8.5). In addition, the main chain carbonyl oxygen atom of His14^Aβ^ accepted a hydrogen bond from the peptide NH of Val2^HC^. This interaction appeared further enhanced by a water mediated interaction between His13^Aβ^ and Ser56 ^LC^.

### Solution NMR analysis with Aβ_1–42_ confirms the X-ray epitope

**Figure 2:**
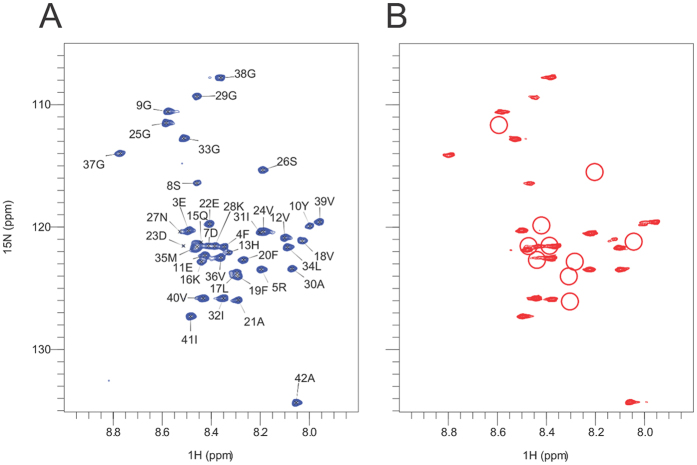
NMR HSQC spectrum of Aβ_1–42_ and CreneFab mapped the epitope. Comparison of the ^1^H/^15^N correlated NMR spectra of ^13^C/^15^N isotopically labeled Aβ_1–42_ (R-Peptide, Georgia), in the absence (**A**) and presence (**B**) of CreneFab. The figures show the region of the resonances belonging to the backbone amide groups. (**A**) ^15^N HSQC spectrum of a 40 μM Aβ_1–42_ solution. The peaks are labeled according to the assignments published elsewhere^40^. (**B**) The same spectrum of Aβ_1–42_ in the presence of a 1:1 ratio of labeled Aβ_1–42_ to unlabeled CreneFab. The red circles show where peaks were broadened such that their intensity was below the noise level.

NMR (nuclear magnetic resonance) is a sensitive method for detection of macromolecular interactions. The 2D ^1^H/^15^N HSQC spectra^39^ of isotopically labeled Aβ_1–42_ are well resolved and have been previously assigned^40^. To confirm the crystallographic observations obtained with a fragment of Aβ peptide, we carried out solution NMR analysis on Aβ_1–42_ in the presence and absence of CreneFab. The ^15^N labeled Aβ_1–42_ peptide was solubilized into the monomeric state as described in the Methods. The ^15^N HSQC spectrum in Fig. 2A shows sharp peaks representing the main chain amides, suggesting the sample was free from aggregation at 40 μM. Upon addition of the CreneFab at a 1:1 molar ratio, we observed differential line broadening (disappearance of peaks) in a subset of the peaks, indicating the residues associated with those peaks either directly interact with the antibody or are located in close proximity of the interaction interface. The peaks corresponding to the epitope from residues His13^Aβ^ to Val24^Aβ^ display significant line broadening as quantified in the methods and indicated in Fig. 2B and Figure S3. Four residues (His6^Aβ^, His14^Aβ^, Asp23^Aβ^ and Asp27^Aβ^) displayed low intensities in the uncomplexed spectra and were not analyzed. Residues immediately adjacent in sequence to the epitope (Glu11, Val12, Gly25, Ser26) also displayed perturbation as expected. None of the residues distal in sequence showed significant line broadening. This observation confirms the epitope identified in the crystal structure is also relevant to full-length Aβ in solution.

### Mutagenesis study reveals binding contribution from critical residues

**Figure 3:**
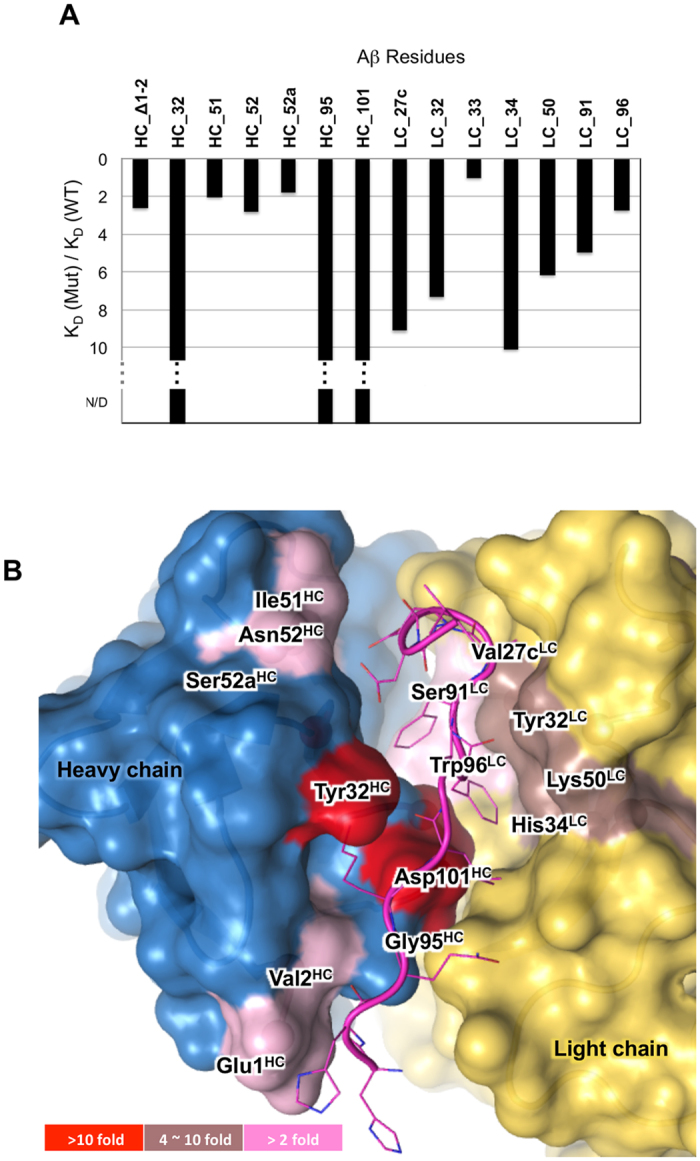
CreneFab alanine mutations impact on affinity. (**A**) A bar graph showing the fold decrease in affinity of individual CreneFab alanine mutants compared to the wild-type determined with SPR kinetic measurements using BIAcore. Mutations at 32^HC^, 95^HC^ or 101^HC^ abolished binding. N/D: not detectable. (**B**) Residues subject to this study are mapped on the crystal structure. The antibody is illustrated in surface presentation. The residues are colored by their impact on binding affinity.

We evaluated binding contributions from Fab residues in contact with Aβ by single alanine mutations. The relative binding affinities measured using surface plasmon resonance (SPR) are shown in Fig. 3, where a 2-fold change of affinity relative to wild-type is considered significant. Alanine mutants of Tyr32^HC^, Gly95^HC^, or Asp101^HC^ completely abolished binding. As described above, Tyr32^HC^ and Asp101^HC^ comprise the Lys16^Aβ^ binding pocket and neutralize its charge (Fig. 1E). The dramatic loss of affinity suggests a proper binding site for Lys16^Aβ^ is indispensible. Gly95^HC^, on the other hand, forms the bottom of the groove. An alanine in this position introduces a steric clash with Phe19^Aβ^ and disrupts the π-π stacking network illustrated in Fig. 1C. Most of the light chain mutations strongly reduced, but did not eliminate, binding. Those residues are in the vicinity of the Phe19^Aβ^/Phe20^Aβ^ binding pocket, including Tyr32^LC^, His34^LC^, Lys50^LC^ and Ser91^LC^. Mutating Ser91^LC^ or His34^LC^ to alanine would disrupt π-π stacking with Phe19^Aβ^. Tyr32^LC^ and Lys50^LC^ side chains form the rim of the binding grove and make contact with the Aβ peptide. Interestingly, the Ser52a^LC^ to alanine mutation only marginally reduced the affinity by 1.8-fold indicating this hydrogen bond contributed a relatively small portion of the total binding energy and a missing hydroxyl group was well tolerated. However, as presented in the discussion section, this interaction contributes to the recognition of Aβ peptide in an extended conformational state. It is noteworthy that the non-conventional antibody- antigen interaction involving the amino-terminus of the heavy chain also contributes to total binding, as deletion of Glu1^HC^ and Val2^HC^ caused a 2.6-fold reduction of affinity. This interaction defines a more extended epitope and potentially distinguishes Crenezumab from other antibodies targeting the middle region of Aβ.

### Electron microscopy showed binding of crenezumab to amyloid fibers

**Figure 4:**
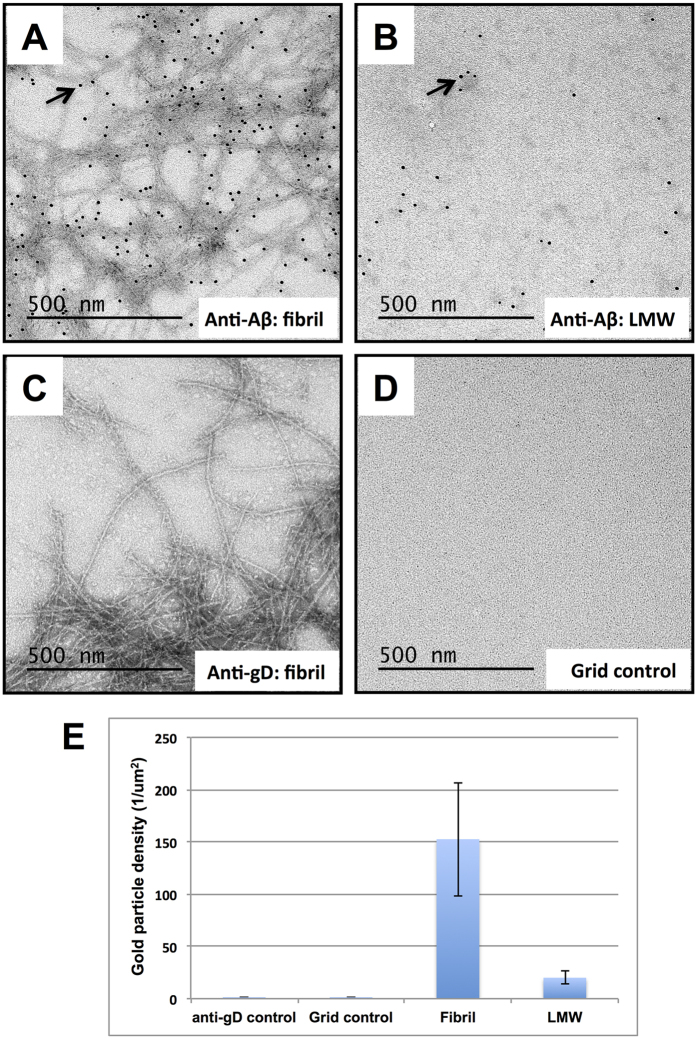
Negative staining immunogold electron microscopy of crenezumab binding to Aβ fibrils. TEM grids were either incubated with a preparation of Aβ fibrils (**A**–**C**) or were left empty as a control for nonspecific antibody adsorption to the grid surface (**D**). Primary labeling was either with crenezumab (**A**,**B** and **D**) or with an anti-gD control antibody (**C**). Detection was with a secondary biotinylated antibody followed by streptavidin conjugated with 10 nm gold particles (**A**–**D**). Arrows point to 10 nm gold particles specifically bound to Aβ fibrils (**A**) or to a low molecular weight (LMW) Aβ aggregated species (**B**). Representative images were taken at 20000× and scale bars are 500 nm. (**E**) Labeling densities were calculated as the average number and standard deviation (STD) of gold particles per 1 um^2^ for N = 20 random areas analyzed: **A** (152.6+/−54.5), **B** (20.1+/−5.9), **C** (0.25+/−0.7) and **D** (0.25+/−0.4).

We have described crenezumab’s unique binding properties, and measured the binding affinities to Aβ monomers and oligomers above. To further characterize the binding behavior of crenezumab with Aβ fibrils, we performed immunogold negative-staining transmission electron microscopy (TEM). We first incubated crenezumab with Aβ_1–42_ fibrils on TEM grids and then labeled the bound crenezumab with a secondary biotinylated antibody, which was subsequently detected with streptavidin conjugated with 10 nm colloidal gold particles (Fig. 4).

By TEM analysis we observed numerous gold particles associated with tangles of Aβ fibrils, indicating binding of crenezumab to Aβ fibrils (Fig. 4A). In addition we found many gold particles bound to low molecular weight (LMW) Aβ aggregated species presumably including Aβ oligomers and small fragments of Aβ fibrils (Fig. 4B). In contrast, gold particles were not observed in the negative control experiments of Aβ fibrils incubated with an unrelated anti-gD antibody (Fig. 4C), confirming that crenezumab bound specifically to Aβ fibrils and to LMW Aβ. In another negative control experiment, we exposed empty TEM grids to crenezumab and all other secondary immunogold reagents and observed no labeling, ruling out the possibility of crenezumab nonspecifically sticking to the grid surface (Fig. 4D). We then quantified the immunogold labeling density by calculating the average number of gold particles per um^2^ area in twenty randomly selected regions in the EM graph. As shown in Fig. 4E, the Aβ fibrils exhibited 7-fold higher labeling density comparing to LMW Aβ, although the same concentration of crenezumab was used for binding in both experiments. The negative control experiments showed near-zero density as expected.

Closer inspection of crenezumab labeled Aβ fibrils revealed a non-uniform distribution of the antibody along the long axis of fibrils (Fig. 4A). This phenomenon suggests that crenezumab is more likely to bind in regions of massive tangling, bending or crisscrossing. We reason that the irregularities in the filament structures may have increased the probability of unfolding individual Aβ hair-pins and consequently exposed the epitopes for crenezumab. Taken together, our TEM studies showed crenezumab binds to specific regions of Aβ fibrils.

## Discussion

The crystal structure of CreneFab with Aβ_11–25_ reveals a well-defined contiguous epitope His13^Aβ^–Val24^Aβ^ in an extended conformation. This region is consistent with the earlier observation by Adolfsson and colleagues^10^. Complete burial of Lys16 and Phe19-Phe20 anchors the Aβ peptide into the CDR groove and explains the high binding affinity. Remarkably, the details of antibody binding seem to hinder Aβ aggregation and promote disaggregation in two ways. First, the antibody occludes half of the “hydrophobic core” sequence responsible for self-association and oligomerization^5^, residues Leu17^Aβ^–Ala21^Aβ^. Second, the hairpin turn is disrupted. It has been shown that a salt-bridge between Asp23^Aβ^ and Lys28^Aβ^ stabilizes the hairpin bend in Aβ self-assembly^28^,^36^,^41^. In our structure, the Asp23^Aβ^ side chain forms hydrogen bonds with Gly33^HC^ and Ser52a^HC^. Hence, sequestration of Asp23^Aβ^ presents a hindrance to aggregation. The important role of Asp23^Aβ^ in crenezumab interaction explains the significant loss of binding associated with the Asp23Ala^Aβ^ mutation (Figure S1). Glu22^Aβ^ has also been implicated in disease^37^. A water mediated H-bond network between Glu22^Aβ^ and crenezumab provide additional hindrance to aggregation (Fig. 1D). Additionally, crenezumab is distinct from those antibodies that recognize a specific Glu22^Aβ^/Asp23^Aβ^ turn conformation^37^, as the crenezumab complex shows no β-turn for these residues.

We measured crenezumab affinities for Aβ monomers and soluble aggregates using a series of single-cycle kinetics titration experiments. The advantage of this method is that it avoids harsh regeneration conditions, which we found to degrade the immobilized Aβ aggregates (data not shown). The soluble aggregates we generated comprised multiple soluble oligomeric species. Data in Figure S2 is therefore a measurement of crenezumab affinity for a heterogeneous population of oligomers. The results are largely consistent, i.e. in the same concentration range, with previously published ELISA results^10^; however, SPR showed a more pronounced affinity distinction in crenezumab affinity for Aβ monomer versus oligomers. The difference in the results from these assay formats is likely a reflection of their different sensitivities to avidity.

**Figure 5:**
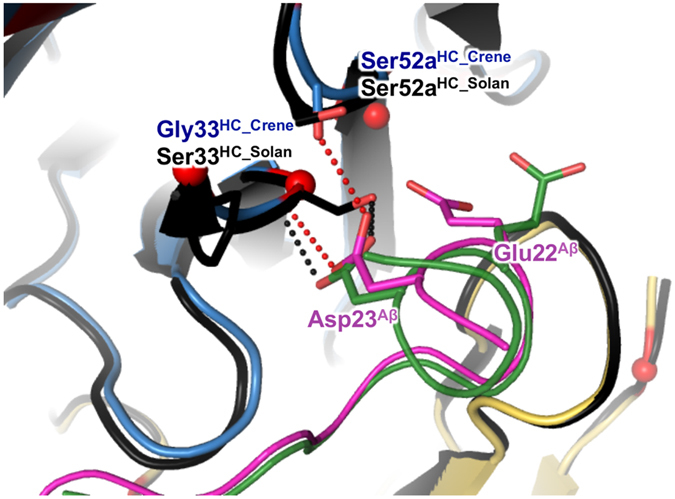
A comparison of crenezumab and solanezumab. Crenezumab/Aβ structure is superimposed onto solanezumab/Aβ structure (4XXD). The color scheme for crenezumab and bound Aβ peptide is same as in Fig. 1A. Solanezumab Fab is shown in black ribbons, and the solanezumab-bound Aβ peptide is in green. The side chains of Glu22^Aβ^, Asp23^Aβ^, and resides at position 33^HC^ and 52a^HC^ of the antibodies are shown in sticks and labeled. The dotted lines indicate hydrogen bond interactions. Black dotted lines are of solanezumab, red dotted lines are of crenezumab. Red spheres indicate amino acid residues different between crenezumab and solanezumab. A more complete view of difference between crenezumab and solanezumab is shown in Figure S2.

Crenezumab and solanezumab both target epitopes in the middle region of Aβ. Despite certain sequence homology in their respective CDRs (Figure S4), they exhibited vastly different specificity for various oligomeric forms of Aβ. This long-standing puzzle was unresolved even after examining the published crystal structure of the solanezumab/Aβ complex^25^. Here, we present a key finding by comparing the two structures. Crenezumab and solanezumab actually target slightly different epitopes, His13^Aβ^–Val24^Aβ^ versus Lys16^Aβ^–Ser26^Aβ^, respectively. While bound to solanezumab, residues Ala21^Aβ^–Ser26^Aβ^ adopt an α-helical secondary structure and residues Ala21^Aβ^–Asp23^Aβ^ make multiple hydrogen bonds and van der Waals contacts with the antibody. Specifically, Asp23^Aβ^ forms two hydrogen bonds with the Ser33^HC_solan^ side chain (Oγ) and main chain (NH) which require that solanezumab binds the α-helical structure. In contrast, crenezumab bound Aβ peptide adopts a random coil structure in the section comprising residues Ala21^Aβ^ through Val24^Aβ^, and is disordered beyond Val24^Aβ^. In the crenezumab complex, residue Asp23^Aβ^ forms a hydrogen bond with the antibody heavy chain residue at position (Gly33^HC_crene^ main chain (NH)) but Glycine offers no side chain for a hydrogen bond like that of the solanezumab Ser33^HC_solan^. Instead, Asp23^Aβ^ forms a unique hydrogen bond with Ser52a^HC_crene^ sidechain (Oγ). This relatively subtle change in the H-bonding pattern supports binding to a more open conformation of Aβ. Comparing the two complexes, the largest Aβ side chain difference is found in Glu22^Aβ^. In binding to crenezumab, Glu22^Aβ^ plunges into a volume surrounded by CDRs H1, H2, L3 and a water cluster. In contrast, the counterpart region in solanezumab is blocked by side chains of Ser33^HC_solan^ and Gln50^HC_solan^, and there are no significant interactions between Glu22^Aβ^ and solanezumab (Fig. 5). On the other hand, at the N-terminal Aβ segment, His13^Aβ^ and His14^Aβ^ make direct interactions with the N-terminus of the CreneFab heavy chain and contribute to the overall binding energy as demonstrated by SPR. We conclude that crenezumab recognizes a non-helical epitope that is shifted by two residues toward the Aβ N-terminus relative to the solanezumab epitope. Only monomeric Aβ peptides have been shown to adopt α-helical structure by solution NMR^42^, albeit in the presence of helix promoting agents such as HFIP or SDS. We propose that the α-helical epitope is present in a subpopulation of monomeric Aβ but is absent in oligomers and higher order aggregates. This provides a plausible reason for solanezumab’s preference for soluble Aβ monomers^20^. Conversely, crenezumab recognizes an epitope in a more extended conformation, which is probably available in a wider variety of Aβ species, including oligomers.

The present studies shed light on the mechanism of action for crenezumab and collectively enhance our understanding of the mechanism behind the various anti-Aβ antibodies currently under clinical investigation. A central question remains for AD therapeutic drug development – what is the nature of target engagement that leads to maximal efficacy in the clinic? Crenezumab is unique in its recognition of a sequence motif and conformation of Aβ that is apparently more broadly available in different aggregation states. Crenezumab buries half of the hydrophobic core and neutralizes the salt-bridge responsible for self-association and thereby destabilizes the β-hairpin, the building block for all forms of Aβ oligomers and fibrils known to date. These characteristics provide a strong rationale for the evaluation of crenezumab as novel potential therapy for AD.

## Methods

### Cloning, expression and purification of Crenezumab Fab

Protease treatment of intact crenezumab produced Fab fragment in low yield, which failed to crystallize. Successful crystallization employed a mutated version of the Crenezumab Fab (CreneFab). It contains the unaltered light chain, one mutation in the VH domain, T108L, and 8 mutations in the CH1 domain. All these mutations are remote from the CDRs and are unlikely to have influenced the structure. To produce CreneFab in *E. coli*, the variable sequences for the light and heavy chain Fab were amplified by PCR using overlapping oligos designed for restriction independent cloning. The product was sub-cloned into *E. coli* expression plasmid AEP1 and transformed into expression strain 24B4. The resulting Fab protein was secreted into the periplasm. The *E. coli* cell pellet was lysed using a cell disrupter (Microfluidics) and the lysate clarified by centrifugation. The Fab was purified from the supernatant by standard protein G column affinity techniques, cation exchange chromatography using SP sepharose, and finally size exclusion chromatography using a Superdex 75 16/60 column. The final protein buffer was 0.15 M NaCl, 20 mM Tris pH 7.5.

### Antibody affinity measurement by Surface Plasmon Resonance (SPR)

A BIAcore^TM^ T200 instrument was used to determine the binding affinity of anti-Aβ Fab by single-cycle kinetics, SPR measurement with Aβ peptides. Briefly, series S sensor chip CM5 was activated with 1-Ethyl-3-(3-dimethylaminopropyl) carbodiimidehydrochloride (EDC) and N-Hydroxysuccinimide (NHS) reagents according to the supplier’s instructions, and streptavidin (Pierce) was coupled to achieve approximately 2000 response units (RU), followed by blocking un-reacted groups with 1 M ethanolamine.

For kinetics measurements, biotinylated Aβ (11–28 or oligomeric 1–42) was first injected at 10 μL/min flow rate into 3 different flow cells (FC) to reach approximately 100–200 RU, except for FC1 (reference). 5-fold serial dilutions of monomeric Fab in HBS-P buffer (0.01 M HEPES pH 7.4, 0.15 M NaCl, 0.005% surfactant P20) from low (0.8 nM) to high (500 nM) were sequentially injected as analyte (flow rate: 30 μL/min) in one cycle with no regeneration between injections. It is difficult to control the density of immobilized Aβ on SPR chips. A kinetic titration series was employed in order to avoid harsh regeneration conditions, which were found to be detrimental to immobilized Aβ aggregates. The sensorgram was recorded and subject to reference and buffer subtraction before evaluation by BIAcore^TM^ T200 Evaluation Software (version 2.0). The association rates (k_on_) and dissociation rates (k_off_) were calculated using a simple one-to-one Langmuir binding model. The equilibrium dissociation constant (K^D^) was calculated as the ratio k_off_/k_on_.

### Generation and Isolation of Aβ Aggregates

Aβ aggregates were prepared from biotinylated synthetic Aβ(1–42) peptides (AnaSpec, Fremont, CA) and initially disaggregated by dissolution in 1,1,1,3,3,3-hexafluoro-2-propanol (HFIP), followed by evaporation under a stream of nitrogen. The peptide film was thoroughly dissolved in 10 mM NaOH, neutralized in an equal volume of 100 mM sodium phosphate buffer, pH 7.4. UV absorbance of the solution at 280 nm was measured, the peptide concentration determined using the respective theoretical extinction coefficient and adjusted by dilution to 1.0 mg/ml with 5 mM NaOH, 50 mM sodium phosphate buffer, pH 7.4.

To generate fibrils for immunogold electron microscopy and soluble, prefibrillar aggregates (oligomers) for affinity determinations, the above material was incubated at 37 °C for ≥48 hours. Insoluble fibrils were removed with a 0.2-μm syringe filter and the filtrate was fractionated by size exclusion chromatography (Superdex 200, GE Healthcare) in a mobile phase of PBS, to isolate soluble aggregate from any remaining monomeric Aβ.

### NMR analysis of antibody Aβ_1–42_ interaction

All NMR spectra were recorded on a Bruker Avance III 800 MHz spectrometer using a TXI cryo-probe. The samples consisted of 180 μL of a 40 μM ^13^C/^15^N labeled Aβ_1–42_ (R-Peptide, Georgia) solution prepared from NaOH- treated stocks as described by the vendor. The final buffer was 20 mM NaPO_4_ buffer set to a pH of 7.0. CreneFab was added to a 1:1 ratio in the same buffer. The samples were measured in 3 mm NMR tubes (Norell, North Carolina) at the calibrated temperature of 300 K. 2D ^1^H/^15^N HSQC spectra were recorded with 2048 data points in the proton dimension and 256 data points in the ^15^N dimension with a total measuring time of 9 hours. All data were processed in TopSpin (Bruker, Karlsruhe) with 4096 data points in the proton and 2048 data points in the ^15^N dimension using forward maximum entropy linear prediction. Due to sample instability, only spectra of peptide alone and in a 1:1 complex with CreneFab were recorded. The ^1^H/^15^N correlated spectra were assigned using the BMRB data base values from submission BMRB-ID 17793 and comparison with published NMR data. Data analysis and visualization was done using CCPN (http://www.ccpn.ac.uk/about). Residues belonging to the binding epitope were identified by signal intensity attenuation and those residues with intensities below the noise mapped to the epitope.

### Immunogold negative staining TEM micrographs of crenezumab binding to Aβ fibrils

Aβ was used after 48 hours of reconstitution (in 5 mM NaOH, 50 mM sodium phosphate buffer, pH 7.4). Formvar and carbon coated TEM grids were incubated for 15 minutes at room temperature with a suspension of reconstituted Aβ fibrils and then blocked for 30 minutes in EM blocking medium for gold conjugates (Aurion) to prevent unspecific sticking of antibodies to the grid surface. The grids were then incubated with crenezumab or anti-gD antibody (negative control) for 45 minutes; all antibodies were diluted 1/200 (final concentration was 5 ug/ml) in EM blocking medium. After washing in PBS the grids were incubated with a donkey anti-human IgG biotinylated secondary antibody (Jackson Immunoresearch; diluted 1/200) for 30 minutes. The grids were then washed in PBS and incubated with a streptavidin-colloidal gold (10 nm) conjugate (British Biocell International) diluted at 1/20 in EM blocking medium for 30 minutes. Finally the grids were washed in PBS, rinsed in distilled water and negatively stained with Nano-W (Nanoprobes) for 60 seconds. The specimens were air dried and then examined in a JEOL JEM1400 TEM at 80 kV. Digital images were captured with a GATAN Ultrascan 1000 CCD camera.

For quantification of gold labeling densities twenty randomly selected areas of each sample were imaged at 20000× and the average number of gold particles +/− standard deviation per 1 um^2^ area was calculated.

### Crystallization, diffraction data collection and structure determination

High throughput crystallization screenings were set up using a Phoenix crystallization robot (Art Robbins, Mountain View, CA) in 96-well format. Optimized apo CreneFab crystals grew as large blocks at 20 °C from hanging-drop vapor diffusion experiments in Linbro plates. The crystallization drops contained equal volumes of protein and reservoir solutions. The final reservoir condition contained 2.4 M ammonium sulfate, 0.1 M HEPES pH 7.5. The apo crystals were preserved for data collection by brief soaking in a cryo-buffer (25% glycerol added to the reservoir solution), followed by sudden immersion into liquid nitrogen.

Complexes of CreneFab and several peptides (Aβ residues 1–42, 15–36, 1–28, 1–16, 11–25, ANASPEC, EGT Group) were produced by mixing solutions of 15 mg of purified Fab and 1 mg of peptide. The best crystals came from the peptide 11–25. Rod-shaped crystals were obtained at 4 °C in Hampton Crystal Screen HT condition A6, which contains 0.2 M magnesium chloride hexahydrate, 0.1 M Tris hydrochloride pH 8.5 and 30% w/v polyethylene glycol 4000. The sitting-drop vapor diffusion experiments were setup using the Phoenix crystallization robot in volumes of 0.5 μL protein sample plus 0.5 μL reservoir solution. The crystals were preserved by soaking into cryo-buffer (30% PEG3350, 0.2 M MgSO_4_, 0.1 M Tris pH 8.5) followed by sudden immersion into liquid nitrogen.

### X-ray diffraction data collection and structure determination

The diffraction data of CreneFab and CreneFab/Aβ complex crystals were collected using monochromatic X-rays at the Stanford Synchrotron Radiation Lightsource (SSRL) beam line 9-2 (MAR325 CCD detector) and beam line 11-1 (Pilatus-6M detector), respectively. In both cases, the rotation method was applied to a single crystal for the complete data set. Data reduction was done using program HKL2000^43^ for CreneFab data, and mosflm and the CCP4 program suite for CreneFab/Aβ complex. Data reduction statistics are shown in Table S1.

The apo CreneFab structure was solved by molecular replacement (MR) using program Phaser^44^. A previously determined Fab structure (PDB: 3R1G, ref. 45) was used as the search model. In order to compensate for possible elbow angle differences, we searched VH/VL and CH1/CL domains separately. Two CreneFab molecules were found in the crystallographic asymmetric unit. Structure refinement was done through iterative manual rebuilding in graphics program COOT^46^ and least-square minimization calculation using programs REFMAC5^47^ and PHENIX^48^. TLS treatment of atomic thermal factors was applied. Several small buffer molecules are identified and built into the final refined structure. Similarly, the CreneFab/Aβ complex structure was determined by MR, using apo CreneFab structure as the search model. There is one molecular complex in the asymmetric unit, and the initial difference map (Fo-Fc) clearly indicated Aβ binding to the antibody. The structure was then refined as for the apo structure. The final refinement statistics are shown in Table S1.

## Additional Information

**Accession codes:** The crystal structures of creneFab and crenFab/Aβ complex are deposited into the Protein Data Bank, accession code 5KMV and 5KNA respectively.

**How to cite this article:** Ultsch, M. *et al*. Structure of Crenezumab Complex with Aβ Shows Loss of β-Hairpin. *Sci. Rep.* **6**, 39374; doi: 10.1038/srep39374 (2016).

**Publisher's note:** Springer Nature remains neutral with regard to jurisdictional claims in published maps and institutional affiliations.

## Supplementary Material

Supplementary Information
