# Supplementary material for: Structure of Crenezumab Complex with Aβ Shows Loss of β-Hairpin
Source: Sci Rep. 2016 Dec 20;6:39374. doi: 10.1038/srep39374 (PMC5171940; doi:10.1038/srep39374)
Supplement: Supplementary Information [file srep39374-s1.pdf]

Structure of Crenezumab Complex with Aβ Shows Loss of beta-Hairpin (Supplemental information)

Mark Ultsch, Bing Li, Till Maurer, Mary Mathieu, Oskar Adolfsson, Andreas Muhs, Andrea Pfeifer, Maria Pihlgren, Travis W. Bainbridge, Mike Reichelt, James A. Ernst, Charles Eigenbrot, Germaine Fuh, Jasvinder K. Atwal, Ryan J. Watts, Weiru Wang

Methods

Epitope mapping by enzyme-linked immunosorbent assay (ELISA)

Epitope mapping was performed by ELISA using two peptide libraries. One contained biotinylated peptides spanning Aβ12-20 (Anawa, Switzerland) and substituting each amino acid by an Ala and the second consisted of biotinylated Aβ13-21, Aβ14-22 or Aβ15-23 and substituting in each case the last amino acid to an Ala, or to a Gly in the case of Ala21. A biotinylated Aβ1-42 peptide was used as a control.

Figure S1 Epitope mapping by Ala or Gly substitutions

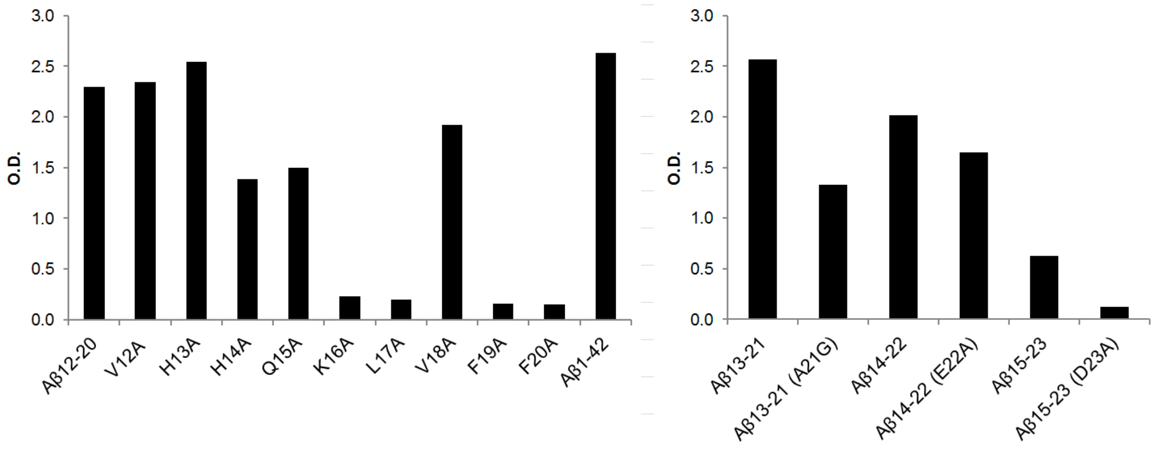

Figure S2 SPR sensorgrams of single-cycle kinetics titration series

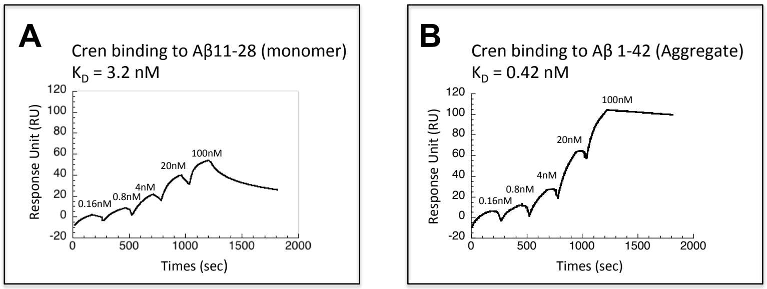

Figure S3 NMR signal intensity modulation by creneFab binding

Bar graph of the relative intensity difference in signal height of A$\beta_{1\text{-}42}$ alone and in a 1:1 molar ratio with creneFab. The trend line shows a moving average of 2 data points. Asterisks indicate residues with peak overlap or of low and noisy signal. These values were omitted in the trend line analysis.

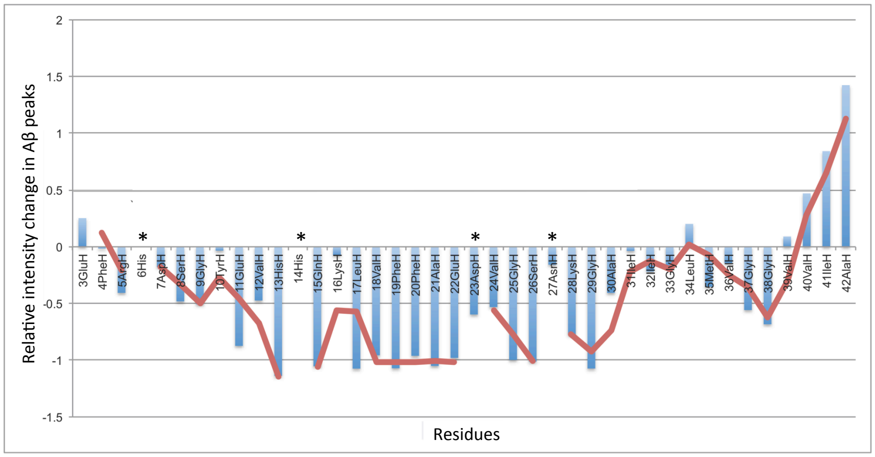

Figure S4 Sequence differences between crenezumab and solanezumab mapped on the creneFab structure. (blue: heavy chain, Yellow: light chain, magenta: Aβ peptide). The red spheres are the Cα atoms of the non-identical residues.

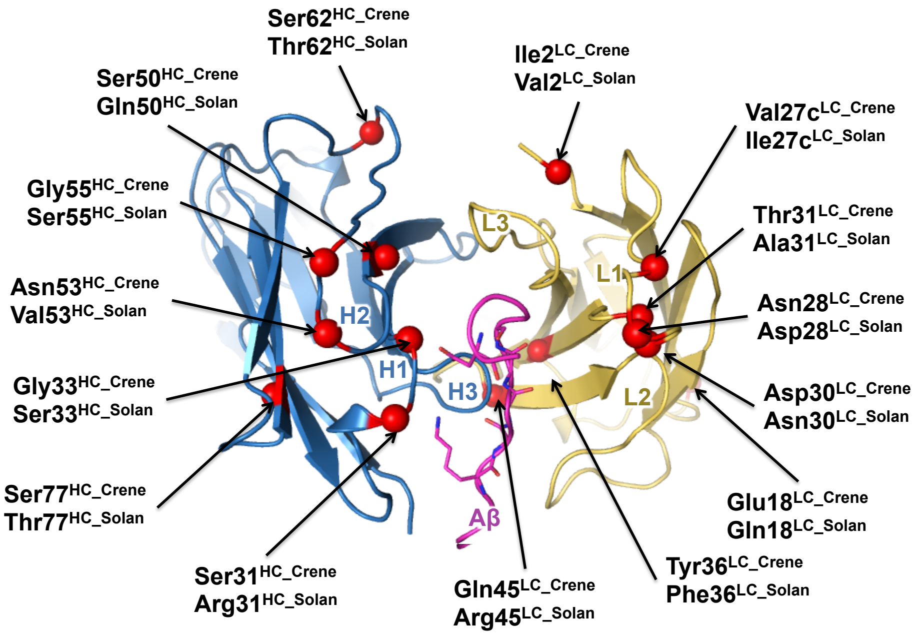

Table S1 X-ray crystallography data processing and structure refinement statistics

	CreneFab/A$\beta_{11\text{-}25}$ complex	CreneFab
PDB code	5KNA	5KMV
Space group	$P2_12_12_1$	$P3_1$
Unit cell	a=44.3Å, b=67.2Å, c=126.8Å, α=β=γ=90°	a=b=129.7Å, c=80.7Å, α=β=90°, γ=120°
Resolution	2.32 Å	2.50 Å
Total measured reflections	18210 (1227) [1]	51884 (5259)[1]
Completeness (%)	98.8 (96.8)	99.1 (100)
Redundancy	4.8 (4.1)	2.9 (2.9)
I/σ	6.9 (2.2)	19.8 (1.7)
Rsym[2]	0.186 (0.586)	0.057 (0.623)
Resolution range	50-2.32 Å	50 - 2.50 Å
Rcryst[3] / Rfree[4]	0.207/0.252	0.183/0.230
Non-hydrogen atoms	3443	6757
Water molecules	142	182
Average B	21.7 $Å^2$	66.0 $Å^2$
r.m.s.d. bond lengths	0.003 Å	0.006 Å
r.m.s.d. angles	0.726°	1.007°
Ramachandran	0.886/0.111/0/0.003	0.870/0.124/0.003/0.003

[1]Values in parentheses are of the highest resolution shell

[2]Rsym = $\Sigma|I_{hi} - I_h|/\Sigma I_{hi}$, where I_{hi} is the scaled intensity of the ith symmetry-related observation of reflection h and I_h is the mean value.

[3]Rcryst = $\Sigma_h|F_{oh} - F_{ch}| /\Sigma_h F_{oh}$, where F_{oh} and F_{ch} are the observed and calculated structure factor amplitudes for reflection h.

[4]Value of Rfree is calculated for 5% randomly chosen reflections not included in the refinement.
